# Multiplexing for *Plasmodium* spp.? Think Again! Comment on Bhowmick et al. Dry Post Wintertime Mass Surveillance Unearths a Huge Burden of *P. vivax*, and Mixed Infection with *P. vivax* *P. falciparum*, a Threat to Malaria Elimination, in Dhalai, Tripura, India. *Pathogens* 2021, *10*, 1259

**DOI:** 10.3390/pathogens11070737

**Published:** 2022-06-29

**Authors:** Nimita Deora, Sonalika Kar, Abhinav Sinha

**Affiliations:** Parasite-Host Biology Division, ICMR-National Institute of Malaria Research, New Delhi 110077, India; nimitadeora0912@gmail.com (N.D.); sonalika@nimr.org.in (S.K.)

The study by Bhowmick et al., 2021 piqued our interest, as it emphasises the importance of using molecular diagnostic approaches to determine true malaria burden, particularly for *P. vivax* and *Plasmodium* mixed-species infections. The study estimated the hidden burden of malaria from febrile and afebrile participants in the dry post-winter months using nested PCR (nPCR). For the benefit of other researchers working in the same field, we would like to raise few cautionary concerns about the modified approach [1] used to screen blood samples in this paper [2], as it was not recommended by the original authors [3,4]. Nested PCR is a well-established molecular approach that is employed to screen *Plasmodium* infections more often than any other method(s). Therefore, it is vital to notify researchers so that they can estimate the real *Plasmodium* burden with more precision in future studies, and avoid possible errors.

In n-PCR for *Plasmodium*, a set of genus-specific (rPLU5 and rPLU6) primers are employed in the first amplification reaction (Nest 1) to amplify DNA fragments from small-subunit ribosomal RNA genes present in the sample from any of the four *Plasmodium* species that infect humans [3,4]. The PCR product of the first reaction is then employed as a DNA template for the second amplification reaction (Nest 2), which uses different species-specific primers to detect sequences within the first reaction’s amplified DNA fragments. In the second stage, it is crucial to employ species-specific primers for each *Plasmodium* species in INDEPENDENT REACTIONS. The authors of the current work cross-referenced Siwal et al., 2018, who attempted multiplexing (*P. falciparum* and *P. vivax* species-specific primers in a single reaction), for nPCR. It should be noted with caution that multiplexing is not a good option using these primers, because it is associated with a significant loss of sensitivity. Snounou et al. observed that if a species is present at a burden 10^2^–10^4^ times less than the other species in a sample, it could be undetected in a multiplex PCR Nest 2 reaction. However, both species were detected when individual reactions were performed for each species. Interestingly, we also found similar results with multiplexing in blood samples collected from febrile patients (manuscript in preparation), as one *Plasmodium* species was found to be completely undetectable in multiplexed reactions as compared to the independent reactions from the same sample; this is a matter of concern, especially in areas of low endemicity.

As quoted from Snounou and Singh, “some sensitivity will invariably be lost as a result of competition between the different amplified fragments for the limited materials present in the reaction” [4]. Therefore, multiplexing is associated with a loss of sensitivity and is unacceptable in low-transmission and low-endemism settings; this is because it may fail to reveal the true proportional burden of different *Plasmodium* species in areas with low parasite loads, even for one of the investigated species. The loss of sensitivity in a multiplexed reaction stems from the intrinsic property of the primers and the same may not be compensated by changing the cycling conditions of the PCR.

In addition, since we cannot estimate the amount of parasite DNA in the total DNA (human and parasite DNA) isolated from human blood, it is not possible to adjust the amount of parasite DNA in the initial DNA template used for NEST 1 in order to compensate for the low parasite load.

In conclusion, PCR multiplexing in a human blood sample actually aims to detect the presence of one or more *Plasmodium* species at the same time, and to reduce the associated costs. Even in a mixed *Plasmodium* species infection, it is highly unlikely that all species are present at an equal parasite load. Thus, multiplexing tends to miss the relatively lesser abundant species which could otherwise be detectable using independent reactions, defeating the entire purpose of multiplexing. This is not only true for the oligonucleotide primers developed by Snounou et al., but has also been reported for other primers [5,6]. Hence, we would like to raise a cautionary flag to the malaria researchers so that this crucial limitation can be overcome in future. 
